# Derivation and Validation of a Prognostic Model for Cancer Dependency Genes Based on CRISPR-Cas9 in Gastric Adenocarcinoma

**DOI:** 10.3389/fonc.2021.617289

**Published:** 2021-02-25

**Authors:** Wenjie Zhou, Junqing Li, Xiaofang Lu, Fangjie Liu, Tailai An, Xing Xiao, Zi Chong Kuo, Wenhui Wu, Yulong He

**Affiliations:** ^1^ Digestive Disease Center, Seventh Affiliated Hospital, Sun Yat-sen University, Shenzhen, China; ^2^ Department of Gastrointestinal Surgery, First Affiliated Hospital, Sun Yat-sen University, Shenzhen, China; ^3^ Department of Pathology, Seventh Affiliated Hospital, Sun Yat-sen University, Shenzhen, China; ^4^ Department of Hematology, Seventh Affiliated Hospital, Sun Yat-sen University, Shenzhen, China; ^5^ Scientific Research Centre, Seventh Affiliated Hospital, Sun Yat-sen University, Shenzhen, China

**Keywords:** Cancer Dependency Map, gastric adenocarcinoma, TCGA, prognostic model, invasion and metastasis

## Abstract

As a CRISPR-Cas9-based tool to help scientists to investigate gene functions, Cancer Dependency Map genes (CDMs) include an enormous series of loss-of-function screens based on genome-scale RNAi. These genes participate in regulating survival and growth of tumor cells, which suggests their potential as novel therapeutic targets for malignant tumors. By far, studies on the roles of CDMs in gastric adenocarcinoma (GA) are scarce and only a small fraction of CDMs have been investigated. In the present study, datasets of the differentially expressed genes (DEGs) were extracted from the TCGA-based (The Cancer Genome Atlas) GEPIA database, from which differentially expressed CDMs were determined. Functions and prognostic significance of these verified CDMs were evaluated using a series of bioinformatics methods. In all, 246 differentially expressed CDMs were determined, with 147 upregulated and 99 downregulated. Ten CDMs (ALG8, ATRIP, CCT6A, CFDP1, CINP, MED18, METTL1, ORC1, TANGO6, and PWP2) were identified to be prognosis-related and subsequently a prognosis model based on these ten CDMs was constructed. In comparison with that of patients with low risk in TCGA training, testing and GSE84437 cohort, overall survival (OS) of patients with high risk was significantly worse. It was then subsequently demonstrated that for this prognostic model, area under the ROC (receiver operating characteristic) curve was 0.771 and 0.697 for TCGA training and testing cohort respectively, justifying its reliability in predicting survival of GA patients. With the ten identified CDMs, we then constructed a nomogram to generate a clinically practical model. The regulatory networks and functions of the ten CDMs were then explored, the results of which demonstrated that as the gene significantly associated with survival of GA patients and Hazard ratio (HR), PWP2 promoted *in-vitro* invasion and migration of GA cell lines through the EMT signaling pathway. Therefore, in conclusion, the present study might help understand the prognostic significance and molecular functions of CDMs in GA.

## Introduction

As the third most common malignant tumor (1), GA has a high mortality rate. Due to the much progress made in diagnosis and treatment over the past few decades, the survival of patients with early GA has improved dramatically (2). It is still an unfortunate fact that the survival of patients with advanced GA remains rather poor, despite the much progress made in chemotherapy, targeted therapies and immunotherapy. The effectiveness of the aforementioned therapies needs to be verified by more further studies (3). Therefore, it is still an urgent task for us to design novel strategies through elucidating the molecular mechanisms giving rise to GA (4).

Initiated by the Broad Institute and the Dana-Farber Cancer Institute, a project named “Defining a Cancer Dependency Map” was performed to discover genes that promoted growth of cancer cells and negatively affected survival of GA patients, the results of which were published in Cell on July 27, 2017 (5). This important study clarified how important some specific genes were to growth and proliferation of human cancer cells. These identified genes might be utilized as targets to develop novel targeted drugs. In this study, more than 500 human cancer cell lines representing 20 different malignant tumors were studied. As cells that can grow infinitely, the aforementioned cancer cell lines were adopted to study the effects of turning off certain genes on proliferation and growth. Cancer cell lines were transduced with a lentiviral vector expressing the Cas9 nuclease under blasticidin selection (pXPR-311Cas9) (6). Subjected to a Cas9 activity assay, each Cas9-expressing cell line was utilized to characterize the effects of CRISPR/Cas9 on these cell lines. Cell lines that were detected with less than 45% measured Cas9 activity were unqualified for further screening. In this study, 769 genes were found to be crucial to survival of cancer cells. Although most of these genes were cancer-specific, about 10% of them were, however, proven to participate in multiple malignant tumors, suggesting their core cellular functions. Although a large number of cancer cell lines were used in their study, the authors along with other researchers pointed out that more further studies were needed to construct a complete map. Workman along with other researchers also stated that cooperation among countries was crucial to a complete cancer dependency map (7). In this study it was also concluded that the best way to predict this dependence was to study gene activity patterns instead of focusing on whether a single gene was defective, which surprised us remarkably (8). With the continuous progress of the research and real-time updates, more related genes were discovered and uploaded to the website https://depmap.org/portal/ (6). In the last update of the dataset, a total of 1,246 genes were included in the analysis and proved to be common and essential in the occurrence and development of various cancers.

In the present study, data of GA were downloaded from TCGA, from which differentially expressed CDMs were identified and the potential functions and mechanisms of these CDMs were explored. A CDM-based prognostic model was also developed and validated as some CDMs could be used as potential prognostic biomarkers.

## Materials and Methods

### Data Processing

Relevant RNA-sequencing and clinical datasets of GA were extracted from the TCGA (https://portal.gdc.cancer.gov/) database. All the downloaded expression profiles were acquired as HT-seq read counts and interpreted using the Ensembl reference database (http://www.ensembl.org/info/data/ftp/index.html). In total, the data of 1,246 CDMs genes were extracted from https://depmap.org/portal/ (6). mRNA that were differentially expressed (DEMs) between GA tissues and normal gastric tissues were identified using the “DESeq2” (9) package of R software. DEMs were defined when adjusted *P*-values <0.01 and log2|fold change| values >0.5 were obtained. The “limma” (10) package of R software was used to normalize the RNA expression profiles and perform variance stabilizing transformation. During the whole process of this present study, the publication guidelines and data access policies of TCGA were abided by. Volcano plots were visualized with the “ggplot2” (11) packages and heatmaps by “pheatmap” (12) packages.

The validation dataset of GC, including the transcription profile based on GPL6947 platform (Illumina HumanHT-12 V3.0 expression BeadChip) and clinical information, were obtained from GSE84437 in GEO (https://www.ncbi.nlm.nih.gov/gds/?term=GSE84437), followed by background correction and quantile normalization using R package “limma”. All mRNA expression values were log2-transformed and standardized for comparability with the TCGA set as above described. The association analyses in the validation study were also conducted using Cox regression models.

### GO Enrichment and KEGG Pathway Analysis

The co-expression of the ten prognosis-related genes was calculated *via* function and pathway enrichment and CDMs were assessed with the Pearson correlation test. In order to minimize false positives, we only included co-expressed Prognosis-related DEMs for function and pathway enrichment analyses when a positive correlation coefficient of >0.3 was achieved. Based on the projection at a specific level of GO terms or KEGG pathways, genes were classified using the “clusterProfiler” (13) package in R software. It was through a hypergeometric distribution with a significance threshold of *P* < 0.05 that functional enrichment analyses were performed for GO terms and KEGG pathways.

### Construction and Validation of Prognostic Models

After deleting patients with incomplete follow-up time and clinical information such as TNM staging, datasets of GA in TCGA-STAD (Stomach adenocarcinoma) were randomly assigned into training cohort or testing cohort. Survival R package was adopted to perform univariate Cox regression analysis on the identified differentially expressed CDMs. And statistical significances of these candidate genes were determined by performing log-rank test.

Subsequently, the variables proven by univariate Cox regression analysis were included in multivariate Cox regression analysis, from which genes with independent predictive ability were verified. The genes with independent predictive ability were used to establish a proportional-hazard regression model, based on which the risk score (RS) was calculated to assess outcomes of patients. The RS formula was illustrated as follow: RS= β1*Exp1 + β2*Exp2 + βi*Expi, in which β stood for coefficient value while the gene expression level was represented by Exp. Patients diagnosed with GA were assigned into low-risk group or high-risk group according to the median RS survival analysis. Overall survival of patients in the two aforementioned subgroups were compared by performing log-rank test. The ‘survivalROC’ package of R software was used to evaluate the predictive capability of the aforementioned prognostic model, where the testing cohort from TCGA database was adopted as the validation group. The prognostic significance of the identified hub CDMs in GA was further verified with the Kaplan-Meier plotter online tool (https://kmplot.com/analysis/) which the main verification datasets are GSE14210, GSE15459, GSE22377, GSE29272, GSE51105 and GSE62254. Ultimately, in order to forecast the likelihood of OS, we established a nomogram with R package, and a statistically significant difference was recorded when the corresponding *P* was less than 0.05.

### Biology Network

To understand the mechanisms and functions of hub CDMs in GA, we created a protein-protein interaction (PPI) network of hub CDMs significantly associated with prognosis under the condition that betweenness was greater than 1 using Cytoscape 3.8.0, utilizing the data from STRING 11.0 (https://string-db.org/). Subsequently, we identified the key modules from the previously constructed PPI network with scores more than 6 and node counts more than 5 as the screening condition using the MCODE Molecular Complex Detection (MCODE) plug-in of Cytoscape software.

### Cell Culture and siRNA Transfection

The gastric cancer cell lines used in our study including AGS, HGC27 were bought from the Shanghai Institute of Cell Biology, Chinese Academy of Sciences (Shanghai, China). After being rewarmed, HGC27 cell line was cultured on dishes filled with RPMI-1640 (Shanghai XP Biomed Ltd., Shanghai, China), while AGS cell line on dished with DMEM/F-12(HAM) medium (Shanghai XP Biomed Ltd., Shanghai, China). Both the two aforementioned culture media were supplemented with 10% fetal bovine serum (FBS; Sage Creation Science Co., Ltd., Beijing, China) and 1% penicillin and streptomycin (Beijing Solarbio Science & Technology Co., Ltd., Beijing, China). The two cell lines were cultured at 37°C in an atmosphere filled with 5% CO_2_ and were detected with no mycoplasma contamination. Guangzhou RiboBio Co. Ltd. (Guangzhou, China) was responsible for designing and synthesizing GenOFFTM si-h-PWP2 siRNA and negative control siRNA oligonucleotides, the sequences of which were presented in **Supplementary Table 2**. Under instructions provided by the manufacturer, we performed the siRNA transfections with 100nm pooled siRNA and riboFECT™ CP Transfection Kit (Guangzhou RiboBio Co. Ltd., Guangzhou, China,18881-1-AP).

### RNA Extraction and Quantitative Real-Time PCR

RNA fast 2000 Reagent (Fastagen, Shanghai, China) was used to extract total RNA from HGC27 and AGS, which was then quantified using the NanoDrop spectrophotometer (Thermo Fisher Scientific, Waltham, MA, USA). Then under the instructions provided by the manufacturer, we reverse-transcribed 1μg of total RNA using a PrimeScriptTM RT Reagent Kit produced by Takara (Dalian, China). Two microliters of cDNA was put into a 20 μl reaction tube to be utilized in quantitative real-time PCR performed by CFX96 Real-Time PCR Detection System (Bio-Rad, Shanghai, China). The PCR primer sequences used in our study were as follows: GAPDH(F) : ACAACTTTGGTATCGTGGAAGG ; GAPDH(R): GCCATCACGCCACAGTTTC ; PWP2(F) : CCACTCGGTACAACGTCAAGT ; PWP2(R) : TCAGGGGAGAAGGACACACTG. All the aforementioned reactions were performed in triplicate without any template control used in each run. The expression of each target gene was standardized with GADPH as the endogenous control and the relative target gene level was determined using the 2-ΔΔCT method.

### Transwell Assay

Twenty-four-well Transwell plates (8 μm pore size; Corning); were used to perform Transwell assays. After PWP2 siRNA or control siRNA was successfully transfected into AGS and HGC27 cell lines, 5×10^4^ cells of both cell lines cultured in 200 μl of serum-free medium were seeded into the upper chamber with the lower chamber filled with 800 μl 10% FBS-supplemented medium. The chamber was then washed using phosphate-buffered saline (PBS) after incubation for 24 h. Afterwards, the residual cells within the upper chamber were removed using cotton swabs while cells having migrated to the lower chamber were fixed using methanol, stained with Giemsa. Then, the stained cells were visualized and photographed using a microscope (Olympus CKX53). The acquired images were then processed using ImageView (X64, version 4.7.14963). For invasive assays, the procedures were basically the same except for 10% Matrigel (BD Biosciences) precoated within upper chamber and doubled number of seeded cells.

### Western Blotting

After being washed using PBS, the cells were lysed with RIPA (radioimmunoprecipitation assay) solution containing a protease inhibitor. The acquired proteins were then quantified using a bicinchoninic acid protein assay (BCA) kit, after which 20 μg of total protein was separated on 8% or 10% sodium dodecyl sulfate polyacrylamide gel (SDS-PAGE) under electric field. The separated proteins were blotted onto a polyvinylidene difluoride (PVDF) membrane (Millipore, USA), which was then blocked with 5% bovine serum albumin (BSA) solution for 1 h and incubated with primary antibodies against GAPDH, E-cadherin, N-cadherin and PWP2 (1:1,000, Proteintech Group, USA) overnight at 4°C. On the second day, the membrane was incubated with secondary antibodies (1:2500) at room temperature for 1 h after three times of washes with TBST (Tris-buffered saline-Tween). After being washed for another three times, bands of conjugate proteins were visualized *via* a ChemiDoc™ MP Imaging system (Bio-Rad Laboratories) with GADPH as the internal control.

### Statistical Analysis

For categorical and continuous variables in testing cohort and training cohort, associations were compared using χ2 test. Candidate CDMs significantly related with OS were statistically confirmed with univariate CPHR (Cox proportional hazards regression) analysis and Kaplan-Meier method. Both significant clinical variables and the risk score formula were determined by stepwise multivariate CPHR analyses. It was through Kaplan-Meier method that survival curves were plotted, which were then tested by the method of log-rank tests. The specificity and sensitivity of the model in predicting patients,survival at each time point were assessed *via* a time-dependent ROC curve. Based on both CDMs and independent clinical variables, a nomogram was constructed *via* multivariate CPHR analyses and validated by plotting C-index and calibration curves. For *in-vitro* functional assays in the three aforementioned independent experiments, all quantitative data were demonstrated as mean ± standard deviation. Differences between the two groups were compared using t-test. The statistical analyses involved in the present study were performed *via* SPSS software (version 23.0), R software (version 3.6.3), or GraphPad Prism 5.0 (GraphPad, La Jolla, CA, USA). A statistical significance was recorded when *P-*value smaller than 0.05 was obtained unless otherwise indicated.

## Results

### Differentially Expressed Cancer Dependency Map Genes in Gastric Adenocarcinoma

**Figure 1:**
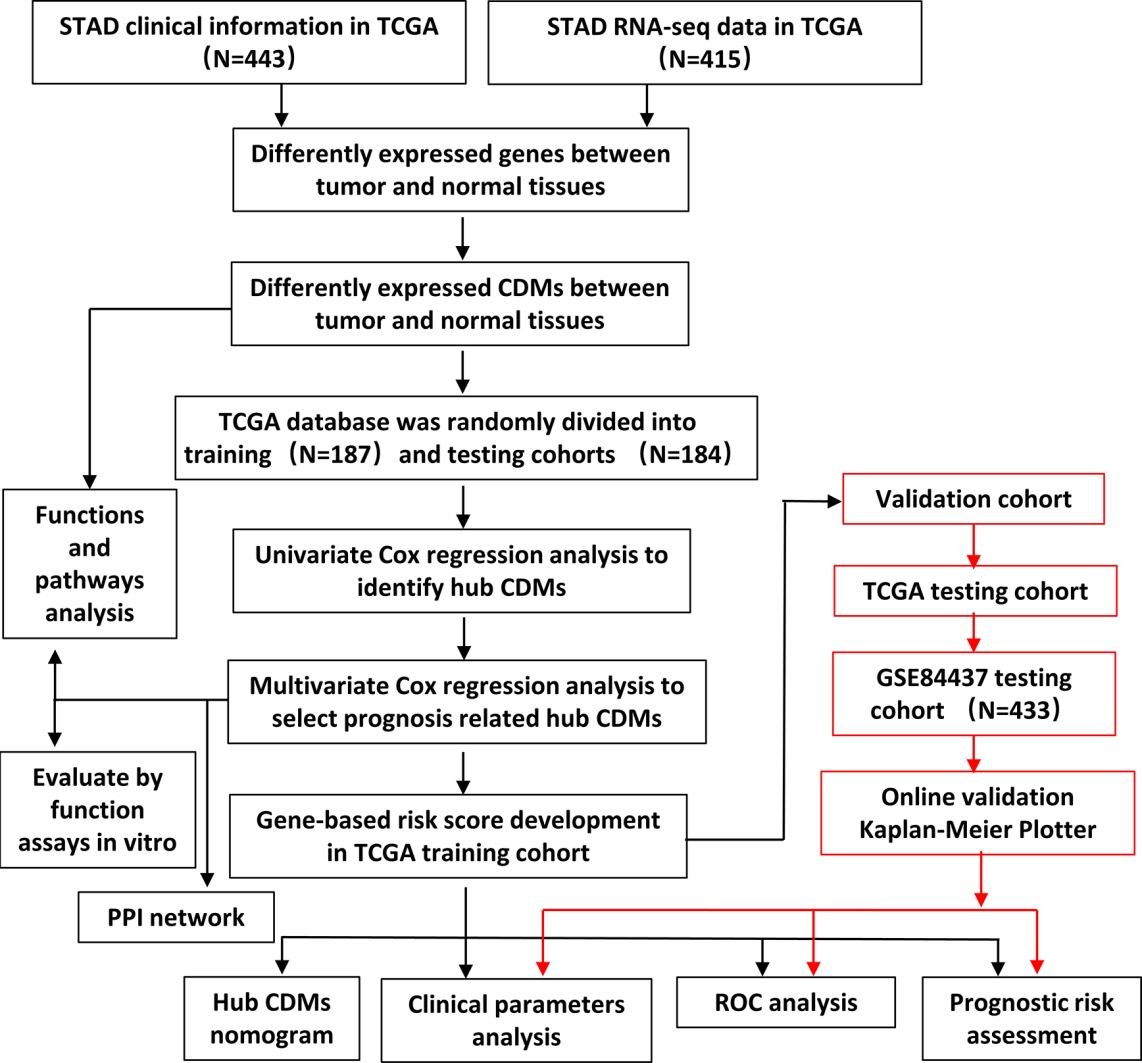
Flowchart of this study.

**Figure 2:**
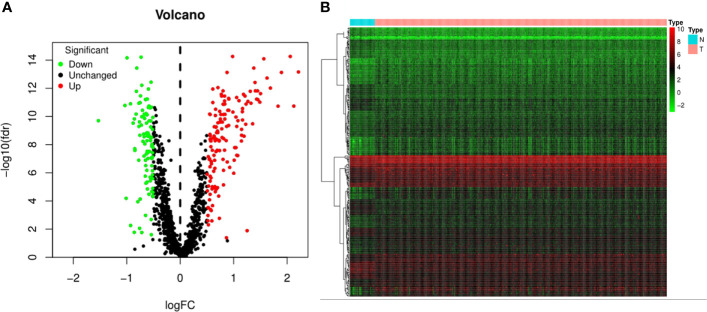
Volcano plot and heatmap of 1,246 Cancer Dependency Maps (CDMs) in Gastric Adenocarcinoma patients from TCGA-STAD Project. **(A)** Volcano plot of 1,246 CDMs in gastric cancer samples from TCGA-STAD Project. Green and red indicated downregulated and upregulated CDMs, respectively. **(B)** Heatmap of 1,246 CDMs in gastric cancer samples from TCGA-STAD Project. Green represents low expression, red represents high expression. N stands for normal tissue and T stands for tumor tissue [FDR(False discovery rate) < 0.05].

The flowchart of the present study was demonstrated in **Figure 1**. After removing genes whose log2|fold change| do not meet statistical significance (*P* > 0.05). It was revealed by “DESeq2” (9) package in R software that among the CDMs in GA, 246 genes were differentially expressed with 147 upregulated and 99 downregulated. After that, we used volcano plot performed by the ggplot2 package to exhibit significantly differentially expressed genes. Then we used the pheatmap package to explore the 246 differential high and low expression genes among the normal and tumor tissue with log2-transformed. (FDR < 0.05, log2|fold change| > 0.5, **Figure 2**).

### GO and KEGG Pathway Enrichment Analysis of the Differentially Expressed Cancer Dependency Map Genes

**Figure 3:**
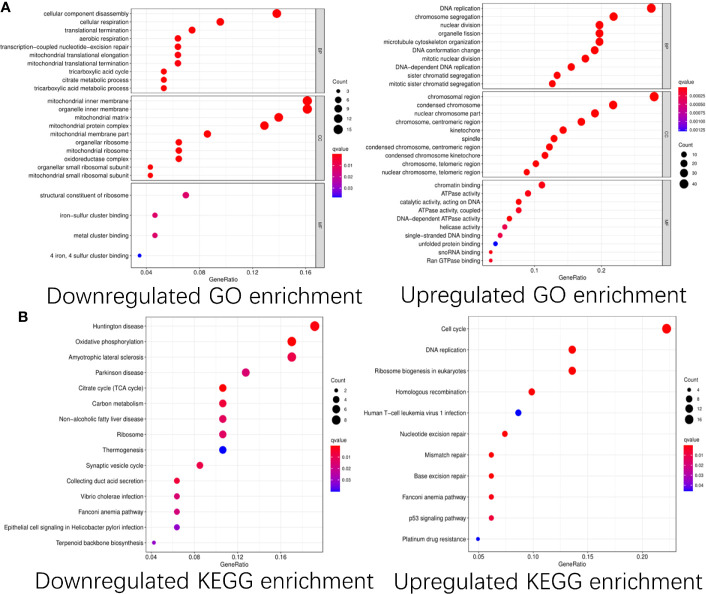
GO and KEGG pathway enrichment analysis of the differentially expressed Cancer Dependency Maps (CDMs). **(A)** GO pathway enrichment of downregulated and upregulated CDMs. **(B)** KEGG pathway enrichment of downregulated and upregulated CDMs.

To explore the functions of these differentially expressed CDMs and mechanisms through which they promoted progression of GA, we performed functional analyses of these downregulated and upregulated CDMs *via* “clusterProfiler” package in R software. As shown in **Figure 3**, significant differences were observed in functional enrichment of downregulated and upregulated CDMs. As for localization within the cell, downregulated CDMs were enriched in related functions of mitochondria and cell energy metabolism. Molecular functional analysis demonstrated that downregulated CDMs participated in structural constituent of ribosome, 4-iron, 4-sulfur metal cluster forming the iron−sulfur cluster. Biological process analysis showed that downregulated CDMs were related to metabolic processes of the whole cell and mitochondrial function, while upregulated CDMs were mainly involved in DNA replication and cell division. As for cellular localization, upregulated CDMs were significantly enriched in centromeric region, condensed chromosome, spindle, kinetochore, centromeric region, nuclear chromosome part, and chromosomal region (**Figure 3A**).

Additionally, as shown in **Figure 3B**, it was also revealed that downregulated CDMs were significantly enriched in synaptic vesicle cycle, thermogenesis, ribosome, carbon metabolism, citrate cycle (TCA cycle), oxidative phosphorylation, non−alcoholic fatty liver disease, Parkinson disease, amyotrophic lateral sclerosis, and Huntington disease while the downregulated ones mainly participated in ribosome biogenesis in eukaryotes, aminoacyl-tRNA biosynthesis, mRNA surveillance pathway, transport and degradation of RNA, and spliceosome.

### Protein–Protein Interaction Network Construction and Key Module Screening

**Figure 4:**
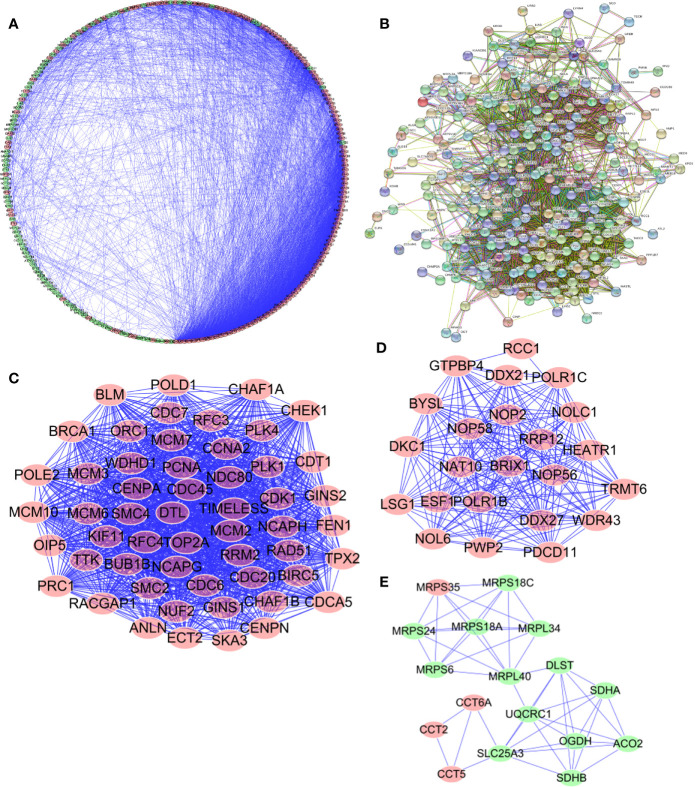
Protein-protein interaction (PPI) network and module analysis. **(A, B)** PPI network for Cancer Dependency Maps (CDMs); **(C)** critical module 1 in PPI network; **(D)** critical module 2 in PPI network; **(E)** critical module 3 in PPI network.

In order to enable us to help us better understand potential molecular functions of these aforementioned differentially expressed genes in GA, we established a protein-protein co-expression network *via* Cytoscape and the STRING database. As shown in **Figure 4**, a total of 246 nodes and 3,377 edges were included in this established PPI network and upregulated genes are marked in red, downregulated genes are marked in green (**Figure 4A**). At the same time, protein-protein interaction of 246 differentially expressed genes was also shown which the number and color of the connections at different points represent the level and quantity of evidence of the connection. In the figure, some nodes have spiral structures inside, which means that the three-dimensional structure of the protein is known, if it is unknown, the nodes are empty (**Figure 4B**). Subsequently, potential critical modules were detected through analyzing the co-expression network *via* the Cytoscape, in which the top three most significant modules were determined. 54 nodes and 1,325 edges were included in module 1 (**Figure 4C**) while module 2 included 23 nodes and 224 edges (**Figure 4D**) and module 3 included 17 nodes and 49 edges (**Figure 4E**). It was revealed by GO and pathway analyses that genes from module 1 mainly participated in DNA replication, chromosome segregation, DNA-dependent DNA replication, DNA conformation change, Human T-cell leukemia virus type 1 infection, oocyte meiosis, and cellular senescence, while genes in module 2 were significantly involved in metabolism, biogenesis, and processing of rRNA, processing and metabolism of ncRNA, ribosome biogenesis in eukaryotes, and RNA polymerase.

### Prognosis-Related Hub Cancer Dependency Map Genes

After 246 differentially expressed genes were shown, to further analyze the effects of CDMs on the prognosis of GA patients, the relationship between the differentially expressed CDMs and OS were assessed through the univariate Cox regression analysis and Kaplan-Meier method, the results of which suggested that 19 candidate hub CDMs were significantly associated with OS through the univariate Cox regression analysis (**Supplementary Table 1**). Afterwards, how these 19 candidate hub CDMs impacted OS were evaluated by Cox multivariate analysis, the results of which demonstrated that ten hub CDMs were independent prognostic predictors for GA patients (**Table 1**).

**Table 1:** Ten Cancer Dependency Maps (CDMs) significantly associated with overall survival in the TCGA-STAD Project.

ID	Coef	HR	HR.95L	HR.95H	*P* value
ALG8	-0.05107	0.950208	0.89973	1.003517	0.066669
ATRIP	-4.24778	0.014296	0.000171	1.192412	0.059837
CCT6A	0.009201	1.009244	1.00174	1.016803	0.015662
CFDP1	0.092187	1.09657	1.03851	1.157876	0.000896
CINP	0.179328	1.196413	0.977263	1.464708	0.082358
MED18	-0.16993	0.843725	0.747413	0.952448	0.006
METTL1	0.080107	1.083403	1.000883	1.172728	0.047503
ORC1	-0.14509	0.864948	0.720126	1.038894	0.120701
PWP2	0.766357	2.151913	1.092021	4.240512	0.026807
TANGO6	0.342845	1.40895	0.977994	2.029809	0.065694

### Construction and Validation of Prognostic Model

**Figure 5:**
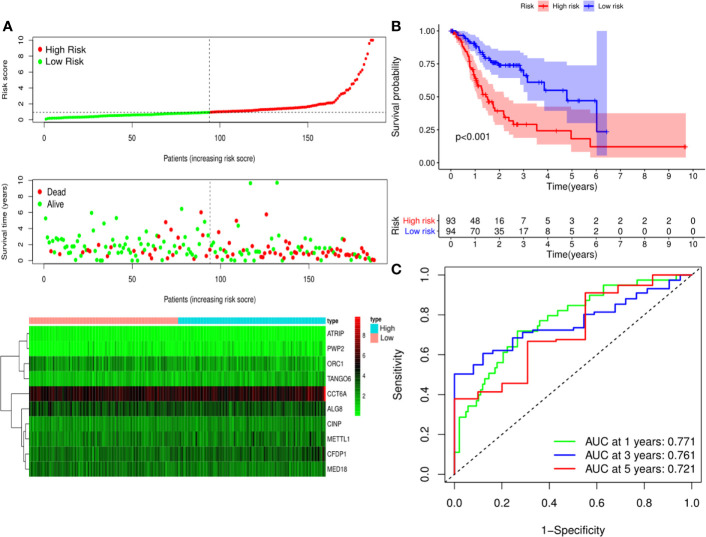
Risk score analysis of ten-Cancer Dependency Maps (CDMs) prognostic model in the TCGA training cohort. **(A)** The risk score distribution, overall survival (OS) status and heatmap of the ten-CDMs signature in the training cohort. **(B)** Kaplan-Meier curves for OS based on the ten-CDMs signature in the training cohort. The tick marks on the curve represent the censored subjects. The number of patients at risk is listed below the curve. **(C)** Time-dependent ROC curve analysis of the ten-CDMs signature for predicting OS in the training cohort.

**Figure 6:**
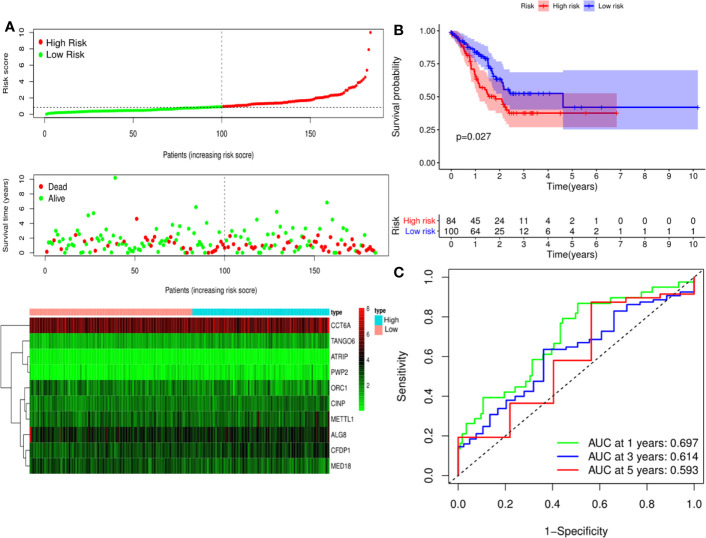
Risk score analysis of ten-Cancer Dependency Maps (CDMs) prognostic model in the TCGA testing cohort. **(A)** The risk score distribution, OS status and heatmap of the ten-CDMs signature in the testing cohort. **(B)** Kaplan-Meier curves for OS based on the ten-CDMs signature in the testing cohort. The tick marks on the curve represent the censored subjects. The number of patients at risk is listed below the curve. **(C)** Time-dependent ROC curve analysis of the ten-CDMs signature for predicting overall survival (OS) in the testing cohort.

**Figure 7:**
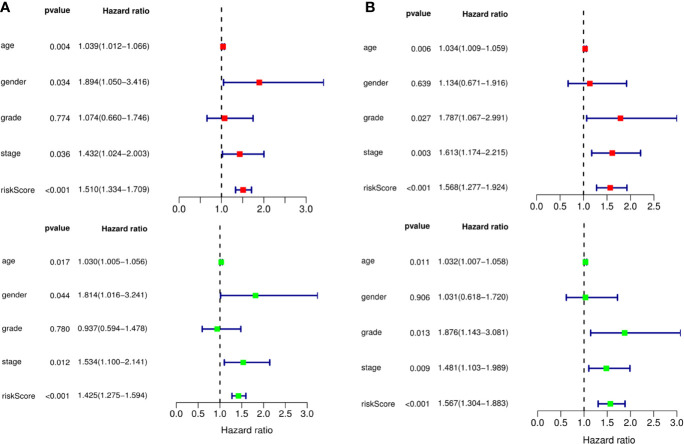
The prognostic value of different clinical parameters. The prognostic value of different clinical parameters. **(A)** Univariate (red) and multivariate (green) COX regression analysis of different clinical parameters in TCGA training cohort. **(B)** Univariate (red) and multivariate (green) COX regression analysis of different clinical parameters in TCGA testing cohort.

A predictive model based on the aforementioned ten hub CDMs was then established. According to the formula: RS (risk score) = (−0.051* Exp ALG8) + (−4.247* Exp ATRIP) + (0.009* Exp CCT6A) + (0.092* Exp CFDP1) + (0.179* Exp CINP) + (−0.170* Exp MED18) + (0.080* Exp METTL1) + (−0.145* Exp ORC1) + (0.766* Exp PWP2) + (0.343* Exp TANGO6), RS of each individual patient was assessed. The predictive capability of RS was evaluated by survival analysis. According to the median RS, the 187 patients from GA TCGA training cohort were assigned into low-risk group and high-risk group. Results of survival analysis demonstrated that compared with those in low-risk group, patients in the high-risk group had significantly poorer OS (p<0.001, **Figure 5B**). In order to further evaluate the prognostic capability of the ten identified hub CDMs, we subsequently performed a time-dependent ROC analysis, results of which revealed that the area under the ROC curve (AUC) of this CDMs RS model was 0.761 at 3 years (**Figure 5C**), indicating moderate diagnostic performance of this model. The survival status of patients, RS and expression heatmap of the signature consisting of ten hub CDMs in the two subgroups were presented in **Figure 5A**. To further verify the validity of the ten CDMs-based predictive model, the 184 GA patients included in the TCGA testing cohort were then analyed, the results of which showed that compared to patients with low-risk score, those with high-risk score had significantly worse OS (*P* < 0.05, **Figure 6B**). As could be seen from **Figure 6C**, AUC of the TCGA testing cohorts were 0.614 at 3 years, justifying its good sensitivity and specificity. The survival status of patients, RS and expression heatmap of the ten hub CDMs in the TCGA testing cohorts were shown in **Figure 6A**. Additionally, the prognostic significance of different variables were assessed among patients of TCGA training and testing cohort by Cox regression analysis, the results of which demonstrated that for both cohorts, both tumor stage and RS could independently predict OS (*P* < 0.05, **Figures 7A, B**). The prognostic values of the ten hub CDMs were also further investigated by GSE84437 cohort (**Figure S1**) and Kaplan-Meier plotter website (**Figure S2**), the results of which revealed that all the ten hub CDMs were not only significantly associated with OS in TCGA but also an independent GEO dataset. In summary, considering all the aforementioned results, we could draw the conclusion that the ten CDMs-based prognostic model was reliable in predicting outcomes of GA patients.

### Building a Predictive Nomogram

**Figure 8:**
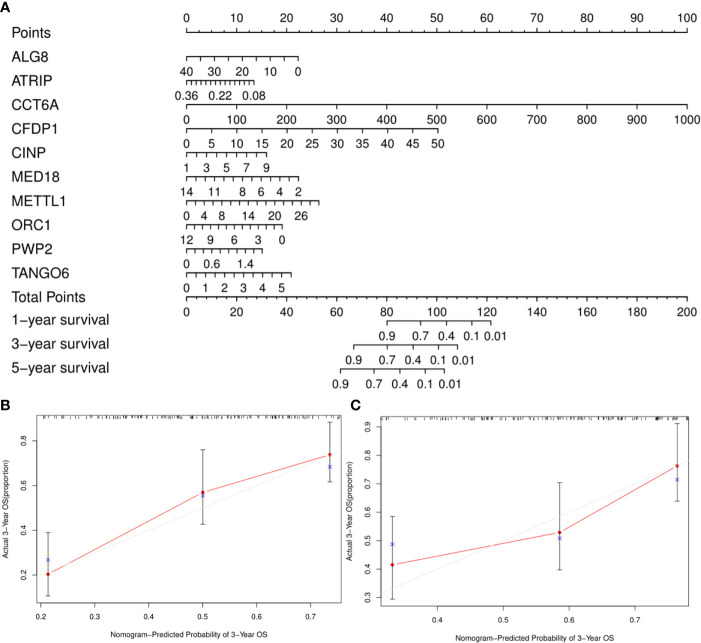
A Prognosis-related Cancer Dependency Maps (CDMs) signature-based nomogram to predict 1-, 3-, and 5-year overall survival (OS) in gastric cancer patients. **(A)** Nomogram for predicting OS. Instructions: Locate each characteristic on the corresponding variable axis, and draw a vertical line upwards to the points axis to determine the specific point value. Repeat this process. **(B, C)** Calibration plots of the nomogram for predicting OS at three years in the training cohort **(B)** and the testing cohort **(C)**. The 45-degree dotted line represents a perfect prediction, and the red lines represent the predictive performance of the nomogram. The blue point represents the actual value, the red curve and the point represent the predictive value, and black marks at the top of the figure represents the position of each patient and their survival status in the model.

A clinically practical model enabling physicians to evaluate survival of patients with GA was then generated by constructing a nomogram with the ten identified hub CDMs (**Figure 8**). According to results of multivariate Cox analysis, each individual variable was given a corresponding point based on the point scale obtained in this nomogram. The point of each variable was determined by drawing a horizontal line. Then the points of all the variables were added up to obtain the patient’s total score, based on which the survival rate of each patient at 1, 3 and 5 years were estimated.

### PWP2 Promoted *In Vitro* Migration and Invasion of Gastric Adenocarcinoma Cells

**Figure 9:**
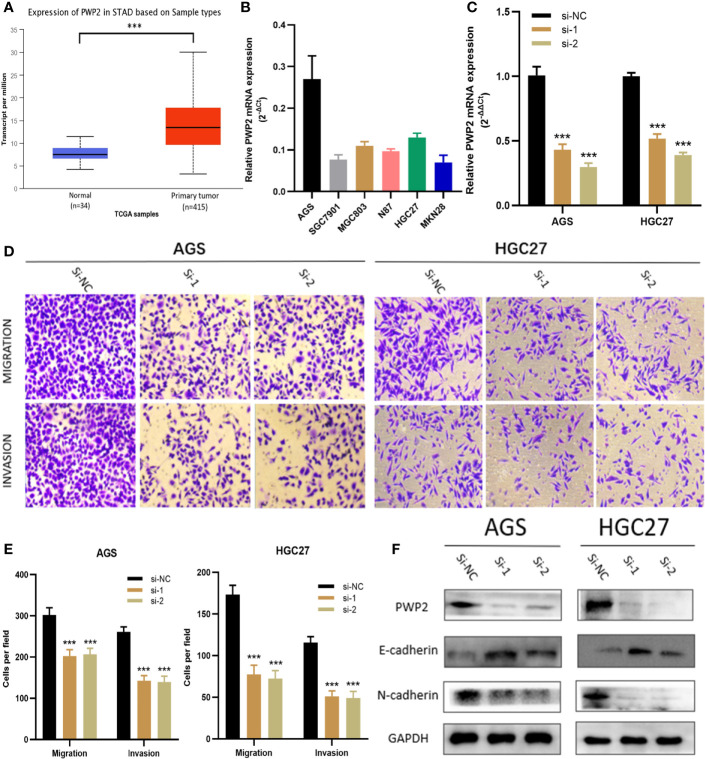
PWP2 enhances the invasion and migration of gastric cancer cells *in vitro*. **(A)** The expression of PWP2 in samples from TCGA-STAD Project. **(B)** Quantitative real-time PCR analysis of PWP2 expression in AGS, SGC7901, MGC803, N87, HGC27, and MKN28 cells. **(C)** Quantitative real-time PCR analysis of PWP2 expression in PWP2-silenced cells and scrambled-siRNA-treated cells. **(D)** The migration and invasion abilities of AGS and HGC27 cells were assessed with Transwell assays after the knockdown of PWP2. (Left panel) Representative images of migration (upper) and invasion (lower) assays. **(E)** The number of cells that migrated or invaded are shown in the histogram. **(F)** The protein levels of E-cadherin, PWP2, N-cadherin were detected by Western blotting in the PWP2-knockdown group. Data are represented as the mean ± standard deviation of triplicate determinations from three independent experiments. Statistical significance was assessed with an unpaired Student’s t-test (two-tailed test). ***P < 0.001.

For this part, whether these identified Prognosis-related CDMs promoted the development and progression of GA was explored. As a tool using CRISPR-Cas9 whole-genome drop out screens to assess dependencies of cancer cells to help guide precision cancer medicines, genetic screens (https://score.depmap.sanger.ac.uk/) was used to identify gastric cancer dependencies. After being assessed for gene fitness effects through CERES (6) which is a computational method to estimate gene dependency levels from CRISPR-Cas9 essentiality screens while accounting for the copy-number-specific effect (**Figure S3**), PWP2 was selected out of these ten Prognosis-related CDMs for further functional assays since it had the greatest impacts on hazard ratio (HR=2.152, P<0.05, **Table 1**) and also appeared in critical module 2 in PPI network(**Figure 4D**). The expression level of PWP2 in GA tissues and normal tissues were evaluated using the data downloaded from TCGA-STAD Project (**Figure 9A**), revealing a significantly higher level in GA tissues than that in normal gastric tissues. A panel of cell lines including AGS, SGC7901, MGC803, N87, HGC27, and MKN28 were cultured and their baseline levels of PWP2 were measured, which revealed greater levels in AGS, MGC803, and HGC27 (**Figure 9B**).

Subsequently, PWP2 siRNA were transfected into AGS and HGC27 cell lines. Then, quantitative real-time PCR analyses were performed, revealing significantly downregulated PWP2 levels in AGS and HGC27 after transfection (**Figure 9C**). It was demonstrated by Transwell assays that the migratory and invasive capabilities of AGS and HGC27 cells significantly decreased after knockdown of PWP2 (**Figures 9D, E**).

As a crucial process during occurrence and progression, epithelial-mesenchymal transition (EMT) was indispensable to invasion and metastasis of GA (14, 15). With the aim of exploring whether PWP2 was involved in EMT, we then quantitatively assessed the expression levels of EMT markers in PWP2-siRNA-transfected GA cells using Western blotting, which demonstrated that the expression level of epithelial marker E-cadherin increased while that of mesenchymal marker N-cadherin decreased (**Figure 9F**). This finding suggested that PWP2 was likely to enhance the migration and invasion of GA cells through promoting the EMT pathway.

## Discussion

It is usually quite difficult for us to predict genes essential for tumor survival, as most malignant tumors originating from epithelial tissues harbor quite many genetic mutations. These genetic mutations are associated with growth of cancer cells and specific vulnerabilities to specific damages. Some of these genetic mutations have been reported to have potential as compelling therapeutic targets (16). The challenge is in finding each targetable vulnerability with the current tools for every cancer (17). Using diverse human cancer cell lines, Aviad Tsherniak and his teammates performed analyses of 501 genome-scale loss-of-function screens (5), in which they developed an analytical framework called DEMETER that segregates on-from off-target effects of RNAi. Scientists dedicating to DepMap by far have profiled hundreds of models based on cell lines to elucidate genomic information and explore cancer cells, sensitivity to genetic and small molecule perturbations. These large-scale databases have been searched for information with the hope of determining genetic targets for developing novel therapies, identifying patients responsive to a certain treatment, and enabling physicians to better understand vulnerabilities of cancer.

The Cancer Dependency Map project has committed to remain open accessed, i.e. under a Creative Commons license making all the data produced by this project freely available to the public. These datasets are released in the form of pre-publication with a quarterly interval and can be downloaded from https://depmap.org/portal/depmap/. As a systematic and novel research method, Cancer Dependency Map has been used to determine the priority targets and drug sensitivity of specific cancer types. And it will ultimately accelerate the process of discovering novel targeted therapies and promote the progress of precise treatment (18).

However, by far, studies on the expression patterns and roles of CDMs are scarce (19, 20). In the present study, based on GA data extracted from TCGA, a total of 246 differentially expressed CDMs were identified. Relevant biological functions and pathways were then comprehensively analyzed. Additionally, it was revealed by univariate and multivariate Cox regression analysis, and ROC analysis that ten hub CDMs were significantly associated with survival of GA patients. Based on the ten identified hub CDMs, a risk model was established in the training cohort and further validated in the corresponding testing cohort. By ROC analysis, this risk model was proven reliable in predicting survival of GA patients. To improve its clinical practicality, we then constructed a nomogram to evaluate survival of GA patients at one year, three years and five years. These findings may enable us to better understand the mechanisms involved in the occurrence and progression of GA and explore new biomarkers for diagnosis and predicting prognoses of GA patients.

It was subsequently revealed by functional and pathway analysis that the upregulated CDMs mainly participated in DNA replication, chromosome segregation, DNA conformation change, DNA-dependent DNA replication and cell cycle pathway while the downregulated ones were mainly involved in cell cycle, mitochondrial inner membrane, organelle inner membrane, mitochondrial matrix and oxidative phosphorylation, citrate cycle (TCA cycle) pathway. Over the past few years, aberrant DNA metabolism (21–23) and DNA processing (24–26) have been reported to play vital roles in various diseases.

To explore the prognostic significance of the ten CDMs, we performed Cox survival analysis for these differentially expressed CDMs among GA patients. A total of ten CDMs that included ALG8, ATRIP, CCT6A, CFDP1, CINP, MED18, METTL1, ORC1, TANGO6, and PWP2 were proven to be prognosis-related. Consistent with our findings, Yi Xuan demonstrated that as a long non-coding RNA, SNHG3 enhanced progression of gastric cancer through regulating methylation of a neighboring MED18 gene (27), and ORC1 is one of the key gene involved in promoting growth, proliferation, and migration of gastric cancer cells (28, 29).

As a cancer with remarkable heterogeneity, gastric cancer is related with various prognoses among different patients (30–32). Therefore, a more sophisticated and reliable prediction model is still urgently needed. At present, a few prognostic models have been established to predict survival of gastric cancer patients, in which, however, the relationship between predicted indicators and gastric cancer has not been clarified (33–35). Subsequently, based on the ten identified hub CDMs, a risk model predicting prognoses of GA patients was established with the TCGA training cohort. It was demonstrated by ROC analysis that this risk model based on the ten CDMs was in selecting out GA patients with poor prognosis, which was further validated by the TCGA testing cohort. The reliability of this risk model described above was also confirmed by Kaplan-Meier analysis. Additionally, revealed by multiple Cox regression analysis of the test cohorts, RS could independently predict prognoses of GA patients. All the aforementioned findings of our study pointed to the clinical practicality of this ten CDMs-based prognostic model. To enable a more intuitive method for the physicians to predict patients’ survival at one year, three years, and five years, we constructed a nomogram.

Responsible for recognizing DNA damage-induced structure and regulating cellular responses to DNA damage and replication stress, ATR-interacting protein (ATRIP) has been proven to interact with MCM complex to promote ATRIP chromatin loading and ATRIP phosphorylation (36). Ying et al. reported that CCT6A could suppress SMAD2 function in NSCLC cells and promote metastasis through TGF-β signaling pathway (37). It was reported by Wu et al. that CINP was identified through genome-wide association and large-scale follow-up analyses as one of the 16 new loci affecting lung function (38). In a study published by Tian et al., METTL1 was reported to be correlated with poor survival of HCC patients and enhance progression of hepatocellular carcinoma *via* PTEN (39). PWP2 belonged to the WD family and was indispensable to the assembly of 90S pre-ribosomal particle. Conditional depletion or decrease of PWP2 protein expression in yeast significantly impaired pre-rRNA processing at sites A(0), A(1), and A(2), resulting in a remarkable decrease in levels of 18S rRNA and 40S ribosomal subunit. It was indicated in the present study that independent of the U3 small nucleolar ribonucleoprotein essential for the initial assembly steps of the 90S pre-ribosome, PWP2 formed part of a stable particle subunit (40). However, roles of PWP2 and the specific mechanisms through which it contributed to GA remain unclear.

Then we accomplished further functional studies on one of the ten CDMs significantly related with OS, PWP2. It was proven by Transwell assays that knocking down significantly reduced the invasive and migratory abilities of GA cells. As a vital contributor to invasion and metastasis of GA cells, EMT was remarkably inhibited after PWP2 was knocked down as the epithelial marker E-cadherin was upregulated while the mesenchymal maker N-cadherin was downregulated, suggesting silencing PWP2 might be utilized to reduce invasion and metastasis of GA cells.

But limitations of the present study are not to be neglected. Firstly, despite the fact that TCGA database has been widely used in various aspects of researches (41, 42), some important preoperative and postoperative parameters such as neoadjuvant chemotherapy, radiotherapy, and immunotherapy were not included, making it impossible for us to perform a comprehensive analyses of these potential parameters. Secondly, the data used in the present study were downloaded from an open-accessed database, making our study retrospective in nature. Thus, further prospective clinical studies are warranted to validate our findings and determine whether the risk model and nomogram can accurately predict survival of GA patients.

In conclusion, we comprehensively investigated the prognostic values and potential functions of differentially expressed CDMs in GA. A risk model that can reliably predict prognosis of GA patients was constructed based on the ten identified CDMs and validated by the TCGA testing cohort. A reliable and clinically practical nomogram predicting prognosis and aiding in individualized management was constructed. Furthermore, the functions of PWP2 in GA was explored, demonstrating that it could promoted invasion and metastasis of GA cells through inducing EMT.

## Data Availability Statement

The datasets presented in this study can be found in online repositories. The names of the repository/repositories and accession number(s) can be found in the article/**Supplementary Material**.

## Author Contributions

YH and WW designed and supervised this study. WZ and JL carried out the bioinformatics analysis. LF, XX, and KH participated in the data analysis and interpretation. JL and TA wrote the manuscript. All authors contributed to the article and approved the submitted version.

## Funding

This study was supported by grants from the National Natural Science Foundation of China (81772579), the Guangdong Province Science and Technology Plan Projects (2014A020212693), “3&3” Project of The First Affiliated Hospital of Sun Yat-Sen University (Yulong He) and Sanming Project of Medical in Shenzhen (SZSM201911010).

## Conflict of Interest

The authors declare that the research was conducted in the absence of any commercial or financial relationships that could be construed as a potential conflict of interest.

## Supplementary Material

Supplementary Table 1 Candidate CDMs significantly associated with the OS of gastric cancer patients.

Supplementary Table 2 The siRNA oligonucleotides against PWP2.

Supplementary Figure 1 Validation the prognostic value of ten hub CDMs in GSE84437 **(A)** Kaplan-Meier curves for OS based on the ten-CDMs signature in GSE84437 dataset. The tick-marks on the curve represent the censored subjects. The number of patients at risk is listed below the curve. **(B)** Time-dependent ROC curve analysis of the ten-CDMs signature for predicting OS in the GSE84437 dataset.

Supplementary Figure 2 Validation the prognostic value of ten hub CDMs in GA by Kaplan Meier-plotter.

Supplementary Figure 3 The PWP2 gene fitness effects **(A)** and gene effect score **(B)** in different gastric cancer cell line.
