# Supplementary material for: Derivation and Validation of a Prognostic Model for Cancer Dependency Genes Based on CRISPR-Cas9 in Gastric Adenocarcinoma
Source: Front Oncol. 2021 Feb 25;11:617289. doi: 10.3389/fonc.2021.617289 (PMC7959733; doi:10.3389/fonc.2021.617289)
Supplement: Supplementary Table 1 — Candidate CDMs significantly associated with the OS of gastric cancer patients. [file Table_1.docx]

| ID | HR | HR.95L | HR.95H | *P* value |
| --- | --- | --- | --- | --- |
| ACTR10 | 1.077041 | 1.018488 | 1.138961 | 0.009261 |
| ALG8 | 1.020685 | 1.005052 | 1.036561 | 0.009324 |
| ATRIP | 0.020004 | 0.001396 | 0.286642 | 0.003979 |
| CCNH | 1.127094 | 1.009708 | 1.258127 | 0.032995 |
| CCT6A | 1.005405 | 1.003021 | 1.007795 | 8.58E-06 |
| CFDP1 | 1.078301 | 1.034907 | 1.123515 | 0.000322 |
| CHAF1A | 0.880974 | 0.817956 | 0.948848 | 0.000818 |
| CINP | 1.187174 | 1.045486 | 1.348064 | 0.008147 |
| COPS2 | 1.063836 | 1.004272 | 1.126932 | 0.035293 |
| DCTN2 | 1.010674 | 1.000911 | 1.020532 | 0.03205 |
| FEN1 | 0.960993 | 0.933956 | 0.988813 | 0.006283 |
| KIF11 | 0.961303 | 0.924649 | 0.999411 | 0.046626 |
| MCM2 | 0.971826 | 0.94752 | 0.996756 | 0.027011 |
| MCM3 | 0.9862 | 0.973983 | 0.99857 | 0.028891 |
| MED18 | 0.903767 | 0.834016 | 0.979351 | 0.013544 |
| METTL1 | 1.052555 | 1.006551 | 1.100662 | 0.024682 |
| ORC1 | 0.895432 | 0.810223 | 0.989602 | 0.0304 |
| PWP2 | 1.889756 | 1.217786 | 2.932517 | 0.004528 |
| TANGO6 | 1.37877 | 1.095879 | 1.734686 | 0.006118 |
