# Supplementary material for: Derivation and Validation of a Prognostic Model for Cancer Dependency Genes Based on CRISPR-Cas9 in Gastric Adenocarcinoma
Source: Front Oncol. 2021 Feb 25;11:617289. doi: 10.3389/fonc.2021.617289 (PMC7959733; doi:10.3389/fonc.2021.617289)
Supplement: Supplementary Table 2 — The siRNA oligonucleotides against PWP2. [file Table_2.docx]

| siRNA |  |  | | |  | | The siRNA oligonucleotides | |  |
| --- | --- | --- | --- | --- | --- | --- | --- | --- | --- |
| genOFFTM st-h-PWP2_001 |  |  | | GCAATAGAGTCACTGTATT | | | | |  |
| genOFFTM st-h-PWP2_002 |  | |  | | | CTGCACCACTTCCACTTCA | |  | |
