# Supplementary figures and images for: Derivation and Validation of a Prognostic Model for Cancer Dependency Genes Based on CRISPR-Cas9 in Gastric Adenocarcinoma
Source: Front Oncol. 2021 Feb 25;11:617289. doi: 10.3389/fonc.2021.617289 (PMC7959733; doi:10.3389/fonc.2021.617289)

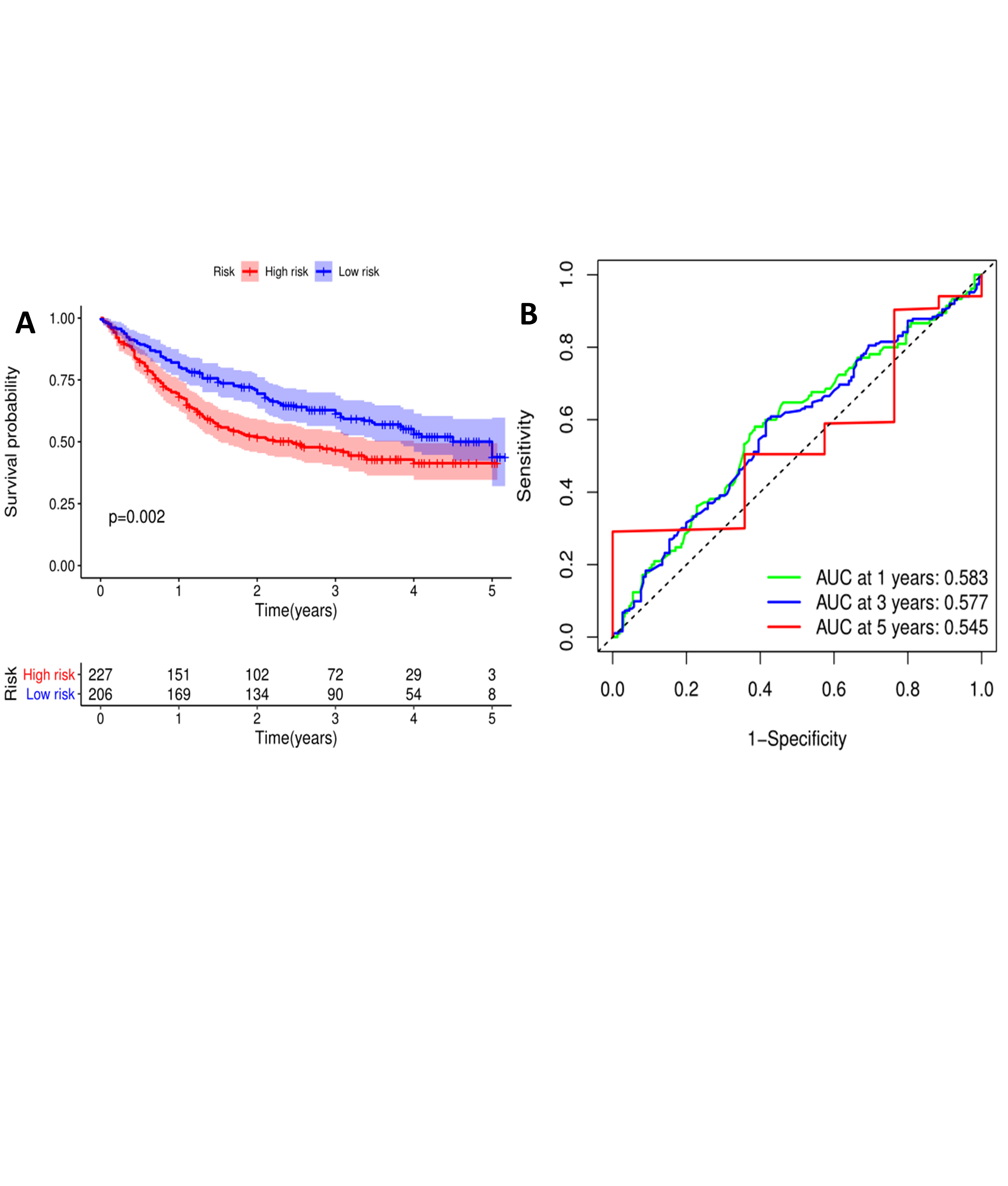

Supplement: Supplementary Figure 1 — Validation the prognostic value of ten hub CDMs in GSE84437 (A) Kaplan-Meier curves for OS based on the ten-CDMs signature in GSE84437 dataset. The tick-marks on the curve represent the censored subjects. The number of patients at risk is listed below the curve. (B) Time-dependent ROC curve analysis of the ten-CDMs signature for predicting OS in the GSE84437 dataset. [file Image_1.tif]

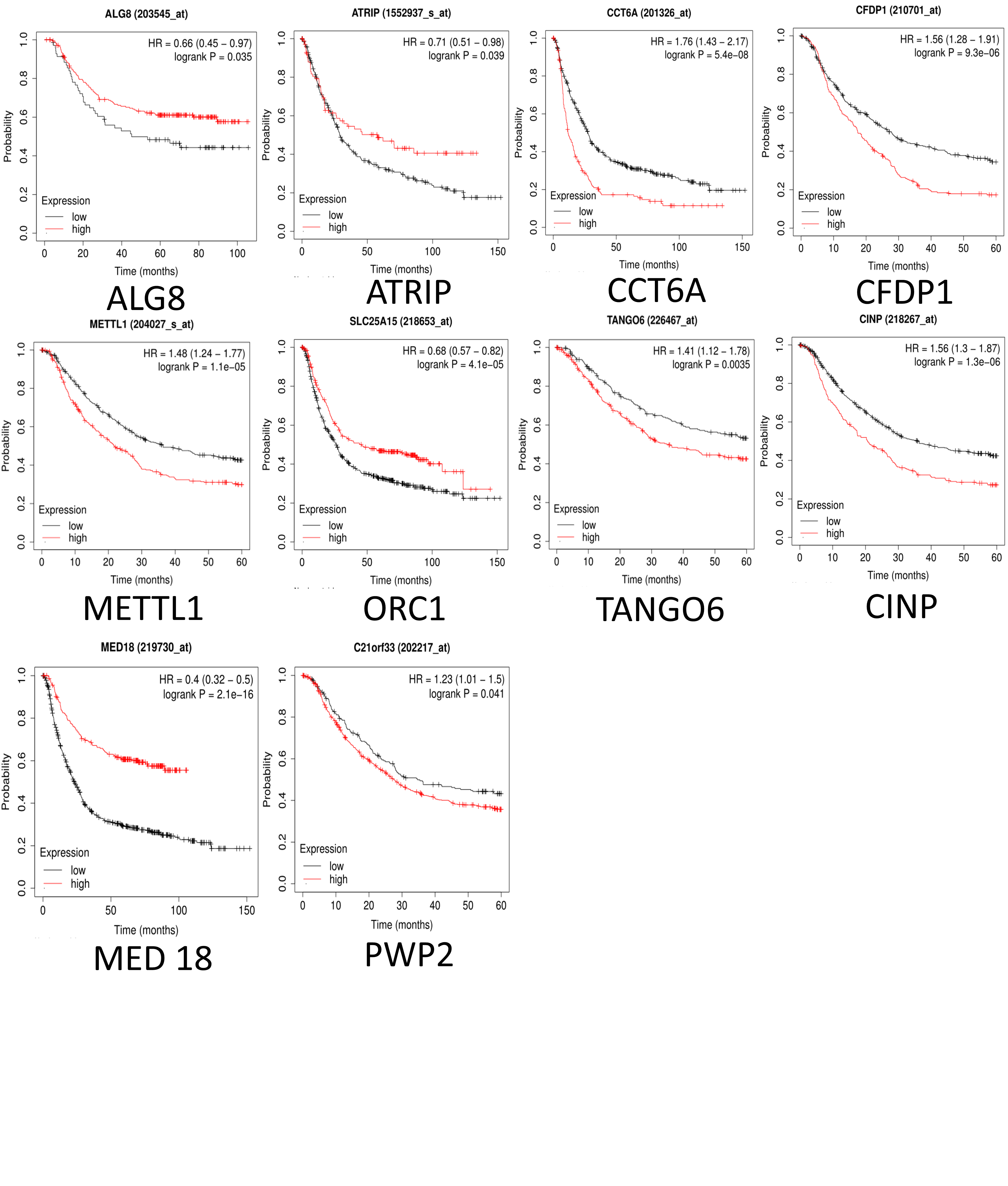

Supplement: Supplementary Figure 2 — Validation the prognostic value of ten hub CDMs in GA by Kaplan Meier-plotter. [file Image_2.tif]

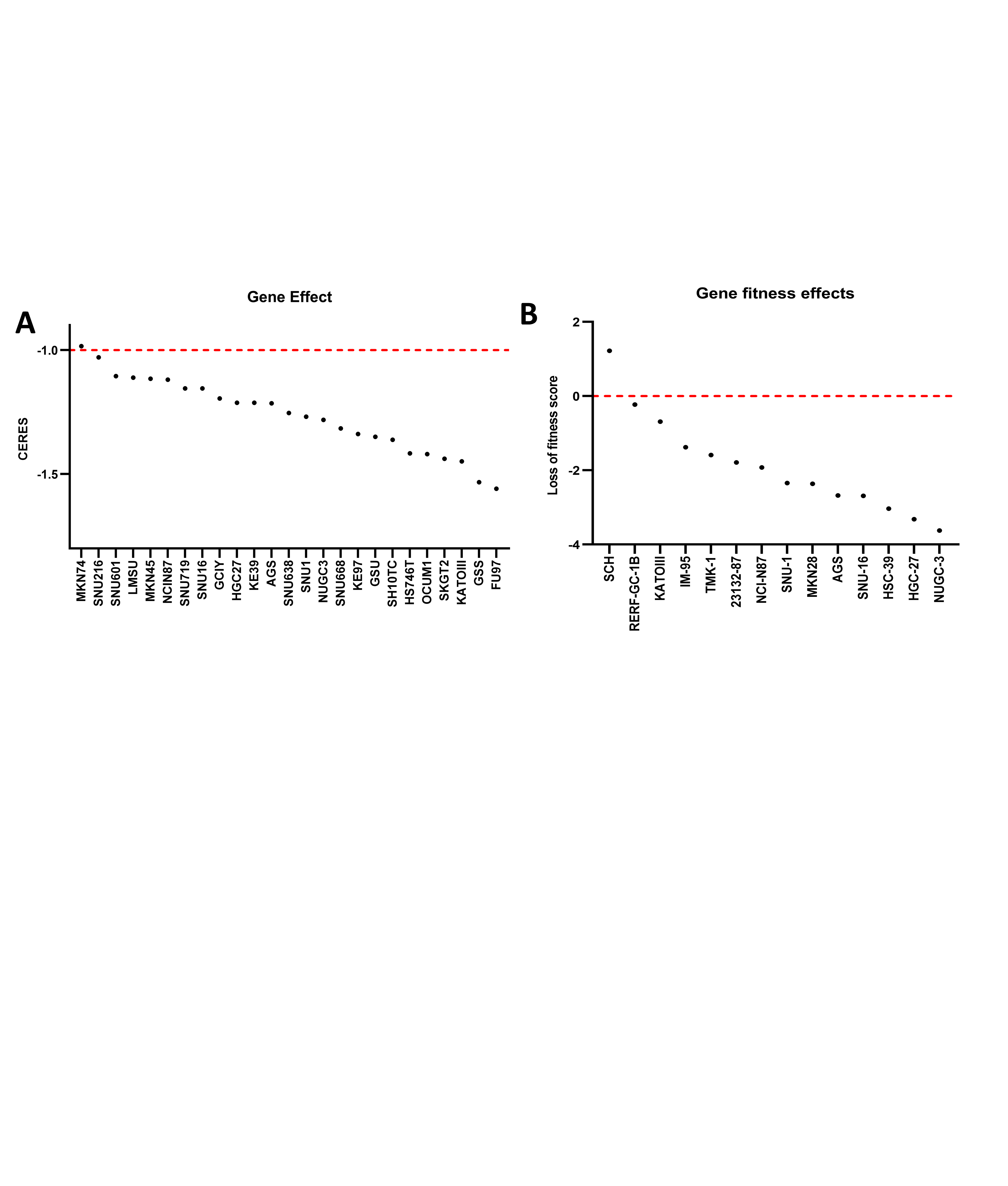

Supplement: Supplementary Figure 3 — The PWP2 gene fitness effects (A) and gene effect score (B) in different gastric cancer cell line. [file Image_3.tif]
